# Influence of study design on digital pathology image quality evaluation: the need to define a clinical task

**DOI:** 10.1117/1.JMI.4.2.021108

**Published:** 2017-06-21

**Authors:** Ljiljana Platiša, Leen Van Brantegem, Asli Kumcu, Richard Ducatelle, Wilfried Philips

**Affiliations:** a Ghent University, Faculty of Engineering and Architecture, Department of Telecommunications and Information Processing, imec-IPI-UGent, Ghent, Belgium; b Ghent University, Faculty of Veterinary Medicine, Department of Pathology, Bacteriology and Poultry Diseases, Merelbeke, Belgium

**Keywords:** digital pathology, image quality, human observer, signal detection, image compression

## Abstract

Despite the current rapid advance in technologies for whole slide imaging, there is still no scientific consensus on the recommended methodology for image quality assessment of digital pathology slides. For medical images in general, it has been recommended to assess image quality in terms of doctors’ success rates in performing a specific clinical task while using the images (clinical image quality, cIQ). However, digital pathology is a new modality, and already identifying the appropriate task is difficult. In an alternative common approach, humans are asked to do a simpler task such as rating overall image quality (perceived image quality, pIQ), but that involves the risk of nonclinically relevant findings due to an unknown relationship between the pIQ and cIQ. In this study, we explored three different experimental protocols: (1) conducting a clinical task (detecting inclusion bodies), (2) rating image similarity and preference, and (3) rating the overall image quality. Additionally, within protocol 1, overall quality ratings were also collected (task-aware pIQ). The experiments were done by diagnostic veterinary pathologists in the context of evaluating the quality of hematoxylin and eosin-stained digital pathology slides of animal tissue samples under several common image alterations: additive noise, blurring, change in gamma, change in color saturation, and JPG compression. While the size of our experiments was small and prevents drawing strong conclusions, the results suggest the need to define a clinical task. Importantly, the pIQ data collected under protocols 2 and 3 did not always rank the image alterations the same as their cIQ from protocol 1, warning against using conventional pIQ to predict cIQ. At the same time, there was a correlation between the cIQ and task-aware pIQ ratings from protocol 1, suggesting that the clinical experiment context (set by specifying the clinical task) may affect human visual attention and bring focus to their criteria of image quality. Further research is needed to assess whether and for which purposes (e.g., preclinical testing) task-aware pIQ ratings could substitute cIQ for a given clinical task.

## 1 Introduction

Currently, traditional microscopy is undergoing a major transformation driven by the development of automated whole slide imaging (WSI),^1^ the technology that is expected to bring critical advancement in pathology diagnostics. The advent of new WSI systems creates the need for a methodology for assessing image quality (IQ) for these systems and for adequate (application-specific) perceptually relevant IQ measures.^2^ Despite the current booming popularity and the advance in new technologies for WSI,^3^ at this moment, there is still no standardization nor formal recommendation for validating the diagnostic quality of digital pathology systems.^4^^,^^5^ In fact, a recent review by Garcìa-Rojo,^6^ which examined international guidelines for digital pathology published in the last eight years, found that most technical aspects are well covered by the guidelines but that they provide limited information regarding IQ and compression. Furthermore, the necessity for developing methodology and building consensus on the evaluation of digital pathology clinical performance has been acknowledged by the FDA’s ongoing research program on the Assessment of Digital Pathology.^7^

In this paper, the main research question is the optimal methodology for evaluating the quality of digital pathology image data. We consider two common approaches for assessing IQ by human observers, explained next: overall-perception-based and clinical-task-based. In the most general approach, human rating of (image) quality is based on a very subjective criterion of the overall impression of quality for which the thresholds may vary considerably from one individual to another. We refer to this kind of quality as perceived overall IQ (pIQ). Alternatively, we can assess the utility of images, i.e., the success rate of performing a specific clinical task when using the images; hereafter clinical IQ (cIQ). Undoubtedly, we expect variability among performances of individuals in the latter approach as well (due to variations in experience, age, instructions provided, and multiple other reasons). However, the cIQ criterion—the level of performance in the task—is not subjective, and it directly reflects the value (utility) of images for their intended purpose. At the same time, cIQ experiments imply not only high costs for recruiting clinical experts as subjects but also substantial time and effort investment for preparing the experiment, especially for selecting appropriate test images.^8^

While the task-based approach to the quality assessment of medical images has been strongly recommended,^9^ it has been meaningfully researched and exploited in a limited number of applications, primarily for x-ray and magnetic resonance imaging (MRI). The dominant task evaluated in the IQ literature is lesion detection, including detection of cancer in breast mammograms or in digital breast tomosynthesis, detection of lung cancer in chest computed tomography images, and detection of multiple sclerosis lesions in brain MRI. Imaging modalities other than x-ray and MRI remain largely ignorant of the task-specific approach and instead rely on the pIQ,^10^ while making the assumption that perceived quality is correlated with clinical performance. In fact, a number of studies continue to this day using pIQ, even in x-ray^11^ and MR imaging.^12^

To date, research and understanding of the relationship between pIQ and cIQ are very limited, creating high risk in using perceived quality to predict clinical performance. The few related studies that have been reported include the work by Taplin et al.,^13^ who considered the task of breast cancer detection in mammography, and by Jiang et al.,^14^ who examined the case of bovine liver tumor detection in MRI. They reported converse findings; the former study demonstrated the lack of correlation between cIQ and pIQ whereas the latter study suggested the presence of correlation between cIQ and pIQ. These two examples illustrate the distinct requirements of individual clinical applications (determined by the clinical task, the anatomy/tissue type, and the imaging modality) that prevent from making generalizations about the preferred approach for evaluating IQ. That is to say, the fact that clinical performance is (not) correlated with perceived quality for one application does not guarantee that the same relationship holds for another application. As Gavrielides et al.^15^ discussed, pooling even clinical image performance across multiple clinical tasks may be misleading as it could mask important image limitations specific to specific tasks. For illustration, even within the context of cancer detection in mammography, an IQ experiment may focus on the detectability of masses and microcalcifications separately, as different types of breast lesions may have different IQ requirements.^16^

As a result, substantial further experimentation and analysis are required. This is especially important for new modalities such as digital pathology, where even only identifying the representative diagnostic task for IQ assessment (IQA) is a nontrivial problem and requires serious considerations and analysis. These range from identifying the most representative clinical problems (abnormalities) to selecting appropriate existing tools for analyzing the collected data and ensuring a sufficiently large test dataset with available ground truth (images with known abnormality), or alternatively developing methods and tools that do not require ground truth, to developing methods for objectively identifying candidate patients (tissue samples/cells) for the IQA study at hand, as argued by Gallas et al.^17^

Recently, Platiša et al.^18^ reported a study that collected pIQ ratings for hematoxylin and eosin (H&E)-stained digital pathology slides of animal tissue samples that were artificially altered to reflect some common factors of image acquisition, management, and display within the WSI system (including blurring, added noise, and JPG compression). The images were assessed by three groups of subjects, according to their expertise profile: expert pathologists, veterinary students, and experts in image processing. Overall, their results suggested that initial criteria for judgment of pIQ in digital pathology images are different for subjects of different expertise profiles, especially between the two expert observer groups; pathologists were less critical of pIQ than imaging experts. On that account, the report concluded that it might be misleading to guide the development of any pathology-specific image algorithms or imaging systems by psychovisual responses of subjects who are not experts in pathology (the target end-users).

In continuation of that research, this paper concentrates on the expert pathologists and presents two new experiments: one that defines a specific clinical task of detecting inclusion bodies under the H&E staining and the other that specifies no clinical task. We collected, in the first experiment, the pIQ ratings as well as the diagnostic performance and, in the second experiment, the ratings of image similarity and image preference. The participating expert pathologists were exactly the same as in Ref. 18. The newly collected data are compared with that of Platiša et al.,^18^ and pros and cons of the different evaluation approaches and protocols are discussed. It is important to emphasize that the experiments are preliminary and limited in sample size (12 reference images representing three sample tissues, as detailed in Sec. 2.1). The main reason behind this limitation is the large number of experiments that had to be performed (see further in Sec. 2.4). Instead, the purpose of this study is to reveal possible interaction between pIQ and cIQ, as well as to make recommendations for the design of future-related studies with human observers. To the authors’ best knowledge, this is the first study with digital pathology images to look into the agreement between the pIQ and cIQ.

Overall, albeit with a very limited image sample size, our results suggest that pIQ assessed without a defined clinical task may not be considered a reliable predictor of cIQ for the studied task and images. A more reliable alternative could be pIQ ratings from experiments in which the clinical task has been specified and used to provide clinical context for the human observer, i.e., to evoke the observer’s appraisal of the clinical relevance of the images while they judge the pIQ.

The paper continues in Sec. 2 with a review of the methodology used for the three human observer experiments (images, participants, protocols, and data analysis). The results of our data analysis are presented in Sec. 3 and discussed in Sec. 4. To conclude, Sec. 5 provides a summary of the main considerations arising from this work.

## 2 Methodology

Prior to our main study, we conducted a prestudy (one experiment only) with four observers for the purpose of selecting the appropriate test parameters for image alterations. For brevity, only the key points of the prestudy are mentioned in this paper without detailed analysis. Then, our main study comprised three experiments, each using the test images of the same pathology samples and each performed by the exact same six pathologists. The images and the observers in the main study were different from those in the prestudy. The three experiments of the main study differed in their specific protocols that define how the images were presented [e.g., single-stimulus (SS) or double-stimuli (DS)], what experimental questions/tasks were asked to the observer to answer/perform and in which order, which rating scales were used, and how/which observer responses were collected. The details are described in the following section.

### 2.1 Images and Alterations

**Fig. 1:**
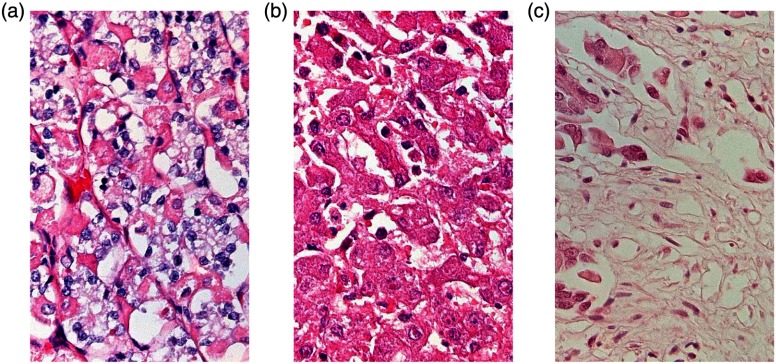
Example reference (M-NONE) images of the three considered tissue samples: (a) gastric fundic glands of a dog, (b) liver of a foal, and (c) gastric fundic glands of a dog. All M-NONE images and their corresponding artificially altered variants (M-Blur, M-Gamma, M-ColSat, M-Noise, and M-JPG) were 1200×750  pixels in size.

The test data set illustrated in Fig. 1 was created from real digital pathology images of animal pathology samples (two samples of gastric fundic glands of a dog and one sample of liver of a foal), each stained with H&E following the same procedure. The images were acquired at 40× magnification using a BX50 Olympus microscope, 3CCD color camera (Olympus DP 50) and saved in TIFF format uncompressed, 24 bit color depth, using “analySIS” software by Olympus Soft Imaging Solutions. Neither compression nor any other image preprocessing was allowed at the acquisition to ensure full control over the extent of image alterations, which we will introduce in Sec. 2.1.2.

The images were potentially showing pathological conditions characterized by inclusion bodies (hereafter lesions). This kind of image is used in the daily routine of veterinary pathologistis to diagnose epidemiologically and clinically important viral diseases in dogs and foals based on the presence of characteristic and disease-specific lesions, i.e., the intracytoplasmic and intranuclear viral inclusions, in the histologic sections.^19^ Specifically, in dogs, the etiology of the disease that was on the pictures was canine distemper virus (CDV), which causes vasculitis, pneumonia, encephalitis, and death; in horses, the etiology of the disease on the slides was equine herpesvirus 1 (EHV-1), which causes respiratory problems, abortion, still birth, or neurological disease. Both CDV in dogs^20^ and EHV-1 in horses^21^ remain highly prevalent; hence, their diagnosis can be considered highly relevant from a clinical and practical point of view. The pathological features that a pathologist uses to visually identify the inclusion bodies are basophilic or eosinophilic globules that are visible with H&E stains and can be found either intranuclear or intracytoplasmatic in the cells (eosinophilic or basophilic depends on the virus, likewise for intranuclear or intracytoplasmic).

#### 2.1.1 Reference images

**Fig. 2:**
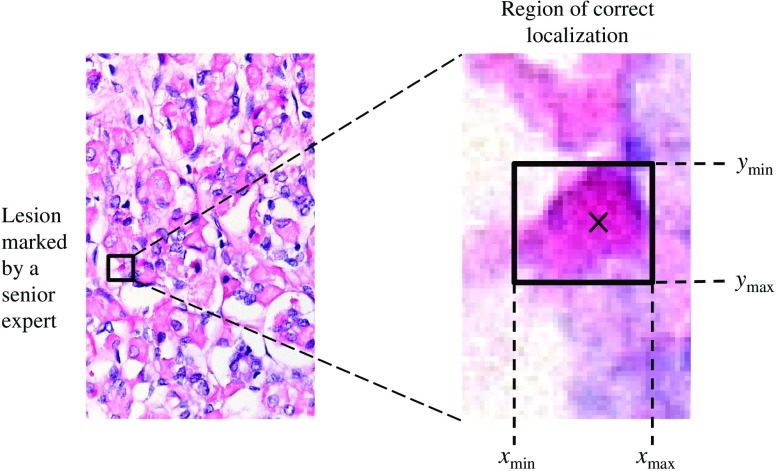
Location-level mark classification. A mark is accepted as “TP” if it belongs to the acceptance region around the actual lesion; otherwise, it is classified as “FP.” The acceptance region is a manually delineated rectangular area determined by the largest width ($x_{\max}-x_{\min}$) and height ($y_{\max}-y_{\min}$) of the actual lesion in the reference image. The actual lesions are the lesions marked by the senior expert with confidence rating above 60%.

First, from the original 5 mega pixel images (2776×2074), we cropped nonoverlapping regions of 1200×750  pixels in size to fit the image presentation requirements (described in Sec. 2.3). The cropped images were considered reference images (hereafter denoted “M-NONE”); see Fig. 2. In total, 24 M-NONE images were used in the main study experiments (eight from each of the three tissue samples); details are in Sec. 2.4.

#### 2.1.2 Image alterations

We were interested in studying the following common factors of image acquisition, management, and display within the WSI system: blurring (possibly caused by thick or folded tissue, incorrect focus, or vibrations during scanning), color, and gamma parameters (typically controlled by the parameters of the display system but also substantial color variation could be due to the process of slide staining or caused by scanning devices), noise (possibly lower for live tissue and higher for dead tissue samples; increasing when the microscope approaches the resolution limit), and image compression (necessary for storage and especially transmission of the very large sizes of digital pathology images).

For the purpose of studying these effects, the reference images were artificially altered by the following nine methods: adding Gaussian blur ($\sigma_{b}=3$), unsharp masking, decreasing/increasing gamma (approx. −5%/+5%), decreasing/increasing color saturation (approx. −5%/+5%), adding low/high-frequency white Gaussian noise ($\sigma_{n}=1.5/\sigma_{n}=10$), and JPG compression (libjpeg^22^ quality 50). The alterations were applied on each reference image and always one at a time. We were not specifically interested in evaluating the levels of the different alterations; therefore, only one level was considered for each alteration adjusted to yield approximately equivalent predicted perceived difference relative to the corresponding reference image—subtle yet noticeable. The degree of predicted perceptual difference was measured in the grayscale (luminance) domain using the high dynamic range visible difference predictor (HDR-VDP).^23^

In a prestudy experiment (see Sec. 2.3), five out of the aforementioned nine image alterations were selected because they had the most prominent effect on the pIQ; these are listed in Table 1. Further in the text, the corresponding five categories of altered (manipulated) images are denoted by an “M-” prefix; for example, we write “M-Blur” to denote an image that was altered by adding Gaussian blur of $\sigma_{b}=3$. Also, we write “M-NONE” to denote a reference image and “M-Any” to refer to any test image but the M-NONE (when the exact type of alteration is not of interest). Thus, there were a total of six images within each reference image (the M-NONE image and its five altered variants).

**Table 1:** Six categories of image alterations considered in the main study.

Image category	Image alteration	Parameter value
M-NONE	None	—
M-blur	Added Gaussian blur	$\sigma_{b}=3$
M-gamma	Decreased gamma	Approx. −5%
M-ColSat	Decreased color saturation	Approx. −5%
M-noise	Added white Gaussian noise	$\sigma_{n}=10$
M-JPG	JPG compression	libjpeg quality 50

Note that the noncolor-aware HDR-VDP mechanism was suboptimal for selecting the levels of alteration in the case of M-ColSat and M-Gamma images, and, therefore, the corresponding results require additional considerations not directly related to the main line of argument in this paper. For example, pathologists are used to some extent of color variation due to staining and tissue age, and the related typical parameters differ among different labs, all of which could lead to larger variation in pathologists’ individual preferences and judgments of the quality of image color. To not distract the reader’s attention, the interpretation and discussion later in the paper will leave out these two image categories. For completeness, the results are shown for all image categories in Table 1.

### 2.2 Participants

A total of six practicing diagnostic veterinary pathologists participated in the main study. Details about their gender, age, and experience distribution are summarized in Table 2. These were different from the four human subjects in the prestudy (one diagnostic pathologist, one veterinary student, and two researchers in image processing). All observers were screened for color vision deficiencies using the Farnsworth Panel D15 test,^24^ and they were all found to not be color blind. The participants were paid for their participation, and they signed a privacy statement that the collected data would be used only in a deidentified form and only for research purposes.

**Table 2:** Distribution of the observers according to gender, age, and experience in diagnostic pathology.

Parameter	Value
Total observers	6
Male observers	1
Female observers	5
Minimum age	25
Maximum age	40
Median age	29.5
Mean years of experience	6.2

### 2.3 Reading Protocols

The three experiments of the main study followed three different protocols for evaluating (reading) images, as summarized in Table 3. Under each protocol, one experiment trial consisted of the observer viewing a given image (pair) and answering all of the questions (observer’s tasks) using their corresponding reporting scales; unanswered questions were not allowed. The methodology for analysis of the collected data is described in Sec. 2.5.

**Table 3:** Three different protocols used in the human observer experiments. Within each protocol, an observer answers the experimental question $Q_{i}$ using its corresponding reporting scale $S_{i}$.

Protocol name	FROC
Stimuli	Reference image and all corresponding altered images (one image per trail)
Visualization	One image viewed individually
Questions	$Q_{1}$: How would you judge overall quality of the image?
	$Q_{2}$: Mark all inclusion bodies in the image and assign them a confidence rating
Reporting scale	$S_{1}$: Continuous [0, 100] [low quality, high quality]
	$S_{2}$: Continuous [0, 100] [low confidence, high confidence]
Data analysis	$Q_{1}$: MdnOS and Kruskal–Wallis analysis
	$Q_{2}$: JAFROC analysis^25^^,^^26^
Protocol name	DS
Stimuli	All pairwise combinations (including self-pairs) of a given reference image and its corresponding altered images (one pair per trail)
Visualization	Two images viewed simultaneously
Questions	$Q_{1}$: How similar are the images?
	$Q_{2}$: Which image do you prefer for overall quality?
Reporting scale	$S_{1}$: Discrete [0, 5] [not similar at all, the same]
	$S_{2}$: Discrete [−3,3] [left image, right image]; includes 0 (no preference)
Data analysis	Median and IQR (per question)
Protocol name	SS
Stimuli	Reference image and all corresponding altered images (one image per trail)
Visualization	One image viewed individually
Questions	$Q_{1}$: How would you judge overall quality of the image?
	$Q_{2}$: How would you judge the level of noise?
	$Q_{3}$: How would you judge the level of blur?
	$Q_{4}$: How would you judge the level of contrast?
	$Q_{5}$: How would you judge the color saturation?
Reporting scale	$S_{1}$: Discrete [0,5] [very low quality, very high quality]
	$S_{2}$: Discrete [0,5] [very disturbing noise, not disturbing at all]
	$S_{3}$: Discrete [0,5] [very disturbing blur, not disturbing at all]
	$S_{4}$: Discrete [0,5] [very poor contrast, very good contrast]
	$S_{5}$: Discrete [0,5] [very poor color saturation, very good color saturation]
Data analysis	MdnOS and Kruskal–Wallis analysis (per question)

The protocol named free-response receiver operating characteristic (FROC, see the top section of Table 3) was the only one including a specific clinical task. After giving their rating of the overall quality of a given image (task-aware pIQ), the observer was asked to mark and rate all suspected color abnormalities (lesions of inclusion bodies), knowing that any number of them is possible, including zero lesions (also called a lesion-free or a normal image). In particular, the observer was asked to mark every suspected location that they considered worthy of mention^27^ and rate their confidence of abnormality using a continuous scale from 0% (low confidence) to 100% (high confidence). The ground truth was determined through image annotations by an experienced senior expert in diagnostic veterinary pathology (see further in Sec. 2.5 and Fig. 2).

Under the DS protocol (midsection of Table 3), the observer was presented with two images simultaneously: one image on the left side and another image on the right side of the screen. The pairs included a comparison of a reference to its corresponding altered image, a comparison of two different alterations of the same reference images, and a comparison of an image (reference or altered) to itself. For each pair of images, there were two rating tasks for the observer: rate the similarity of the two images (in terms of IQ) and rate the preference between the two images. The similarity was rated using a six-point discrete scale from 0 (not similar at all) to 5 (the same) while the preference rating was done on a seven-point discrete scale from −3 (prefer left image) to +3 (prefer right image), allowing also a zero (0) response value (no preference).

Finally, the SS trials with hidden reference ^28^ as described in ITU-R Recommendation BT.500-13^29^ displayed a single randomly chosen image at a time (bottom section of Table 3). For each image, the observer performed five tasks rating different IQ attributes: pIQ, perceived blur disturbance, perceived quality of contrast, perceived noise disturbance, and perceived quality of color saturation. Each attribute was rated using a six-point absolute category rating scale^29^ ranging from 0 to 5. The better (less disturbing) the perceived attribute, the higher the score. Note that in this paper only the pIQ data will be of interest; the data collected under $Q_{2}$ to $Q_{5}$ have been presented in another publication by Platiša et al.,^18^ which has already been referred to in Sec. 1.

### 2.4 Experiments

The experiments reported in this work are summarized in Table 4. The prestudy was performed in the pilot experiment following the SS protocol in which four observers viewed 50 images (5 reference and their 5×9 artificially altered images), and the goal was to select five (out of nine) image alterations with the most dominant perceptual effects. The three experiments of the main study each followed one of the three aforementioned protocols: exp1, FROC protocol; exp2, DS protocol; and exp3, SS protocol. They were designed to each answer a specific research question with respect to the quality of color digital pathology images:

• Exp1: What is the effect of image alterations on clinical performance?
• Exp2: How sensitive are pathologists to image alterations, i.e., which alterations are perceptually salient for them?
• Exp3: How do pathologists judge IQ and its attributes?

**Table 4:** Overview of the experiments performed in the prestudy (pilot) and in the main study (exp1, exp2, and exp3).

Name	Protocol	Context	No. observers and observer experience	Image data	Trials per observer
Pilot	SS	Technical	One diagn. pathologist, one veterinary student, and two imaging experts	5 reference images and their 5×9 altered versions	50
Exp1	FROC	Clinical	Six diagn. pathologists	12 reference images and their 12×5 altered versions	72
Exp2	DS	Technical	Six diagn. pathologists	21 pairwise combinations with repetition within three reference images	63
Exp3	SS	Technical	Six diagn. pathologists	12 reference images and their 12×5 altered versions	72

The same six observers performed all three main study experiments, always in the same order: exp1, exp2, and exp3. The intention was to first allow the pathologists to do what they are trained for and most familiar with (the diagnostic task in exp1), then ask them to judge images according to their personal criteria (image similarity and preference in exp2), and only at the end introduce them to some more technical terminology (noise, blur, contrast, and color saturation) and ask them to rate the specific attributes of IQ (in exp3). All experiments used a fully crossed study design, i.e., all observers viewed all images/pairs. Since in exp1 the observers were asked to perform a clinically relevant task, this experiment has a very obvious “clinical” context. In the other two experiments, however, the observers’ tasks were exclusively about the quality of the images, and, except for the image content itself (pathology tissues), there was no mention of the clinical context whatsoever; we refer to this context as “technical.” The notion of context will be of interest later when we discuss the results of our experiments.

The images used in these experiments were as follows: (exp1) 12 reference images (4 crops from each of the three tissue samples) and their 12×5 artificially altered images, (exp2) 3 out of the 12 reference images from exp1 (1 crop from each of the 3 samples, as shown in Fig. 1) and their 3×5 artificially altered images, and (exp3) a separate set of 12 reference images (4 different crops from each of the 3 samples) and their 12×5 artificially altered images. Thus, the exp2 dataset was a subset of the exp1 dataset, whereas the dataset of exp3 was different. This is because the investigation originally included only exp1 and exp2 and participants naive to diagnostic pathology. Later on, diagnostic pathologists were involved. To allow comparison with the previous results, the datasets for exp1 and exp2 were kept the same. However, since the main purpose of recruiting pathologists was to conduct task-based image assessment, the images for exp3 were selected to fit the requirements of an FROC experimental paradigm. As described in Sec. 2.1, all 24 reference images represent the same three pathology samples (8 reference images from each of the three samples). Presentation of the (pairs of) images was randomized for all experiments.

The images were displayed on a 3MP medical color LCD display (MDCC-3120-DL, Barco N.V., Kortrijk, Belgium) with the color management set to fidelity. No image adjustment (zoom or window level) was allowed. The observers were seated at 50 cm from the display and were allowed to lean back and forth. The experiments were conducted in a controlled viewing environment to ensure consistent experimental conditions: low surface reflectance and approximately constant ambient light. There was no time limitation.

Prior to the experiments, participants were asked to fill out a profile questionnaire concerning their age, gender, profession, and experience. Each experiment was conducted in a separate session, often split by a few days. The session started with a brief written introduction to the purpose of the experiment and explanation of the protocol. Prior to the experiment, there was a brief training without feedback including 5 to 10 trials to clarify the procedures, to familiarize the participants with the user interface, and to ascertain that the rating scale was properly understood. The images used in the training trials were different from the images in the experiment trials. At any point before the first experiment trial, observers could ask questions. After completing the last trial, there was a short questionnaire about the experience with the experiment.

### 2.5 Data Analysis

The main method of data analysis used with each protocol is indicated in Table 3. The analysis was performed based on the pooled measurements for all test images within a given experiment.

Overall, the categorical data were analyzed with median and interquartile range (IQR) and Kruskal–Wallis testing for the statistical significance. By comparing the collected data of individual observers among each other through visual inspection of scatter plots, no inconsistent observers were identified. Then, given the non-Gaussian descriptive statistics of our collected data, we have chosen to use the median opinion score (MdnOS) and the (25%, 75%) IQR to evaluate observer ratings. To test for differences among MdnOSs, we perform the Kruskal–Wallis nonparametric one-way analysis of variance. We note that the data in our experiments do not always comply with the condition of data independency that is required for the Kruskal–Wallis analysis, i.e., the altered images in our experiments are manipulated variants of the reference images, and thus M-Any images are related to M-NONE images. Nevertheless, for simplicity reasons and given the preliminary character of the study, we will consider the images of different M-groups (approximately) independent. We also perform posthoc pairwise comparisons at a significance level α=0.05.

In the case of FROC protocol in exp1, the collected human data consist of an arbitrary number of mark-rating pairs per image. Under the FROC paradigm, a marked (suspected) location is classified as a correct lesion localization (true positive, TP) if the mark falls within an acceptance region of the actual lesion; otherwise, it is a wrong lesion localization (false positive, FP). In our study, the acceptance region was defined as a rectangular area determined by the largest width and height of the actual lesion in the reference image (annotated by an experienced senior expert in diagnostic veterinary pathology); for illustration, see Fig. 2.

For computing the FROC figure of merit (FOM), we used the jackknife AFROC (JAFROC) method proposed by Chakraborty and Berbaum^25^ and later refined by Chakraborty.^26^ The FOM takes values in the range 0 to 1, where FOM=1 corresponds to a prefect observer who marked every true lesion (TP) and did not mark any normal image (number of FPs is zero). The data were analyzed using the freely available JAFROC software.^30^

## 3 Results

We present here the results of data analysis in terms of the following: (1) pathologists’ performance in localized lesion detection ($Q_{2}$ from exp1), (2) pathologists’ subjective judgment of image similarity and preference (exp2), and (3) pathologists’ subjective judgment of the overall IQ ($Q_{1}$ from exp1 and $Q_{1}$ from exp3). As explained at the end of Sec. 2.1.2, the results are presented for all image categories listed in Table 1 while the interpretation and discussion leave out the M-ColSat and M-Gamma images.

### 3.1 Clinical Image Quality

Table 5 reviews the parameters of the diagnostic task performed by the observers within $Q_{2}$ from exp1. Using the terminology of FROC studies, the observers are referred to as readers, the test images as cases, and the six different categories of image alteration (reference included) as treatments.

**Table 5:** Parameters of the JAFROC experiment.

Name	Value
No. readers	6
No. treatments	6
No. normal cases	5
No. abnormal cases	7
Fraction normal cases	0.417
Min lesions per image	1
Max lesions per image	2
Mean lesions per image	1.286
Total lesions	9
Mean nonlesion localization marks per reader on normal images	2.667
Mean nonlesion localization marks per reader on abnormal images	1.091
Mean lesion localization marks per reader	0.722

**Fig. 3:**
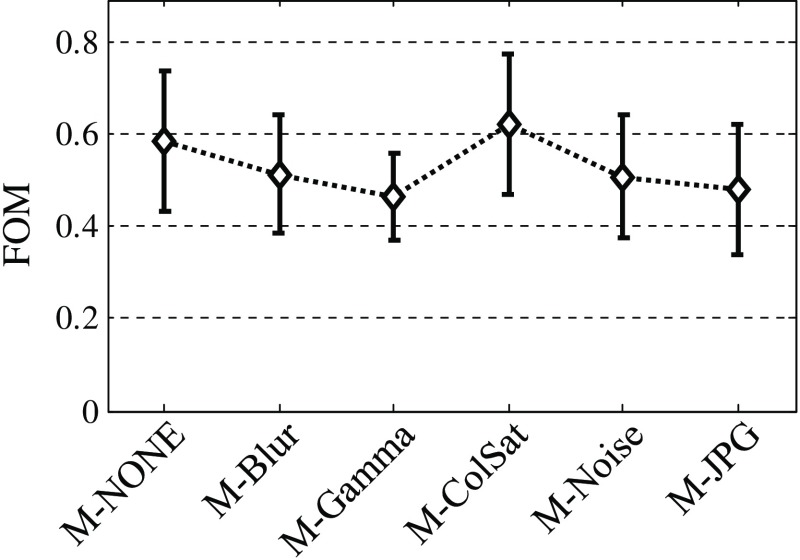
JAFROC FOM for all considered types of image alteration (including M-NONE). The FOM is averaged over observers and the error bars correspond to 95% CI.

The results of the JAFROC analysis are summarized in Table 6 and graphically represented in Fig. 3. Overall, the null hypothesis that the six categories of image alterations yield equivalent performance in the considered diagnostic task (joint detection and localization of lesions) is rejected at a α=0.05 significance level [F(5,25)=4.95, p=0.0028]. In general, JAFROC suggests using at least 50 image samples (cases) for the findings to be generalizable to the population of images (random-case analysis) and more than three observers to generalize to the population of observers (random-observer analysis). Otherwise, the analysis is valid only for the specific images/observers used in the study (fixed-case/fixed-observer analysis). Given the very low number of test cases in our experiment (12 cases per treatment, see Table 5), we did not attempt to generalize to cases and we reported random-reader fixed-case analysis only. Based on our experimental data, JAFROC predicted that (minimum) 53 cases would be required to conduct random-case analysis [assuming six readers, significance level α=0.05, effect size 0.05, desired statistical power (1−β)=0.8].

**Table 6:** Difference in FOM between all pairings of image alterations (including M-NONE) and the corresponding 95% confidence intervals (CIs). The asterisk symbols indicate statistically significant differences in FOMs.

Compared image alterations	Difference in FOM	95% CI
M-Blur versus M-Gamma	0.05000	[−0.03106,0.13106]
M-Blur versus M-ColSat	−0.10926	${\left[-0.19032,-0.02820\right]}^{*}$
M-Blur versus M-Noise	0.00556	[−0.07550,0.08661]
M-Blur versus M-JPG	0.03333	[−0.04773,0.11439]
M-Blur versus M-NONE	−0.07037	[−0.15143,0.01069]
M-Gamma versus M-ColSat	−0.15926	${\left[-0.24032,-0.07820\right]}^{*}$
M-Gamma versus M-Noise	−0.04444	[−0.12550,0.03661]
M-Gamma versus M-JPG	−0.01667	[−0.09773,0.06439]
M-Gamma versus M-NONE	−0.12037	${\left[-0.20143,-0.03931\right]}^{*}$
M-ColSat versus M-Noise	0.11481	${\left[0.03376,0.19587\right]}^{*}$
M-ColSat versus M-JPG	0.14259	${\left[0.06153,0.22365\right]}^{*}$
M-ColSat versus M-NONE	0.03889	[−0.04217,0.11995]
M-Noise versus M-JPG	0.02778	[−0.05328,0.10884]
M-Noise versus M-NONE	−0.07593	[−0.15698,0.00513]
**M-JPG versus M-NONE**	−0.10370	${\left[-0.18476,-0.02265\right]}^{*}$

As seen from Table 6, statistically significant differences among the levels of task performance were found only for M-NONE to M-JPG comparison (marked in bold in the table); other five comparisons involve either M-ColSat or M-Gamma images and will not be discussed further.

### 3.2 Similarity and Preference Judgments

The similarity judgments collected under question $Q_{1}$ from exp2 are summarized in Table 7, and the preference data of $Q_{2}$ are shown in Table 8. Both tables present the median values computed over pooled ratings by all observers for all image pairings within a given category of alterations (M-NONE, M-Blur, and so on). As an indicator of variance, we show the corresponding IQR, the difference between the first and the third quartiles.

**Table 7:** Summary statistics of image similarity ratings collected in exp2 under question “How similar are the images?” while using a six-point Likert-type scale from “not similar at all” (0) to “the same” (5). For each image-pairing, the median and the IQR of the similarity ratings are shown.

	M-NONE	M-Blur	M-Gamma	M-ColSat	M-Noise	M-JPG
M-NONE	5.00 [4.00,5.00]					
M-Blur	3.00 [1.25,3.75]	4.00 [4.00,5.00]				
M-Gamma	4.00 [4.00,4.75]	2.00 [1.00,3.75]	5.00 [4.00,5.00]			
M-ColSat	1.00 [0.25,2.00]	1.00 [0,2.50]	1.00 [0,1.75]	5.00 [4.25,5.00]		
M-Noise	5.00 [4.00,5.00]	4.00 [2.00,4.00]	4.00 [3.00,5.00]	1.00 [0.25,1.00]	4.50 [4.00,5.00]	
M-JPG	4.50 [4.00,5.00]	3.50 [2.00,4.00]	4.00 [4.00,4.75]	1.00 [0.25,1.75]	4.00 [4.00,5.00]	5.00 [4.00,5.00]

**Table 8:** Summary statistics of image preference ratings collected in exp2 under question “Which image do you prefer for overall quality?” while using a seven-point Likert-type scale from “left image” (−3) to “right image” (+3). In the table, the left image side corresponds to the columns and the right image side is represented in the rows. For each image-pairing, the median and the IQR of the preference ratings are shown.

	M-NONE	M-Blur	M-Gamma	M-ColSat	M-Noise	M-JPG
M-NONE	0 [0,0]					
M-Blur	2.50 [2.00,3.00]	0 [−0.75,0.75]				
M-Gamma	0 [−1.00,1.00]	−2.00[−3.00,−2.00]	0 [0,0]			
M-ColSat	2.00 [−1.00,2.00]	−1.50[−3.00,1.00]	2.00 [−1.00,3.00]	0 [0,0]		
M-Noise	0 [0,0.75]	−2.50[−3.00,−1.00]	0 [0,1.00]	0 [−2.00,2.00]	0 [0,0.75]	
M-JPG	0 [0,0.75]	−2.00[−3.00,−1.25]	0 [−1.00,1.00]	−0.50[−2.00,2.00]	0 [−1.00,0]	0 [0,0]

First, looking at the diagonal elements of Tables 7 and 8, we note that most pairs of identical images (self-pairs) are correctly judged (the highest similarity scores 5 or 4, and the neutral preference scores around 0), which suggests that the observers correctly understood their tasks and were mainly doing the right thing. Then, we turn to the intercategory image comparisons. Again correctly, for all image pairs rated high in similarity, the preference ratings remained close to 0 (no preference between the two images). The only exception is the comparison between M-Blur and M-Noise rated high in similarity (median [IQR]=4[2,4]) but with preference for M-Noise (−2.5[−3,−1]). Excluding M-ColSat and M-Gamma images, some dissimilarity was perceived only with M-Blur images, judged less preferred compared to M-NONE (3[1.25, 3.75]) and M-JPG images (3.5[2, 4]).

### 3.3 Perceived Overall Image Quality

**Fig. 4:**
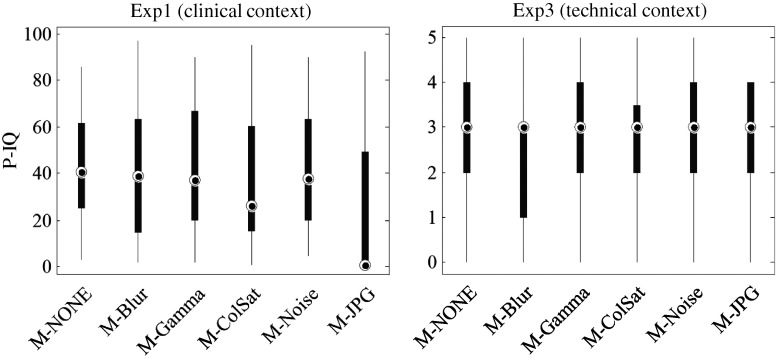
Overall IQ ratings by diagnostic pathologists: (a) results from exp1 (task-aware pIQ), continuous rating scale from 0% to 100% and (b) results from exp3 (conventional pIQ), discrete six-point rating scale from 0 to 5. For both experiments, a higher rating score corresponds to higher pIQ. In both plots, the x-axis represents the type of image alteration (M-NONE, M-Blur, M-Gamma, M-ColSat, M-Noise, and M-JPG). Each box in the plot indicates the median, the IQR, the 1.5 IQR interval (whiskers); no “outliers” (measured points outside of the whisker range) have been identified.

Figure 4(a) shows the task-aware pIQ ratings collected under $Q_{1}$ from exp1 (continuous scale 0 to 100, clinical context), and Fig. 4(b) represents the conventional pIQ ratings from $Q_{1}$ from exp3 (absolute category scale from 0 to 5, technical context). Note that, overall, the ratings from exp1 fall in the lower range of the rating scale (lower part of the y-axis) compared to the ratings from exp3. Based on the data collected in exp1, pathologists found JPG-compressed images to be significantly different from all other treatments (Kruskal–Wallis test, p<0.001); all other pairwise comparisons among treatments (alterations) were not significant. In exp3, however, all treatments were equivalent except M-Blur was found significantly different from M-Gamma (Kruskal–Wallis test, p<0.01). The results are further discussed in Sec. 4.

## 4 Discussion

Pathologists’ responses collected in the clinical-related experiment of our study ($Q_{2}$ in exp1) suggest that the diagnostic FROC performance (cIQ) is significantly lower for the images with the lossy JPG compression (introducing some typical artifacts such as blockiness) compared to the considered reference images. The fact that compressing images could cause degradation in the diagnostic performance (due to often lower confidence ratings of the marked abnormalities) suggests that this level of JPG compression may not be acceptable for clinical digital pathology, that is, in particular, for the diagnosis of inclusion bodies under the H&E staining. Clearly, this indication should be verified with a more extensive study (more observers, more images of specific tissues, and additional especially lower levels of JPG compression) before any final conclusions can be made.

Previously, Marcelo et al.^31^ found no statistically significant difference between the diagnostic accuracy of noncompressed and that of JPG-compressed images in telepathology. The same was concluded by Seidenari et al.,^32^ although they noted the intraobserver reproducibility in the diagnostic judgment to be lower for compressed images. In the domain of image analysis, Nicolosi et al.^33^ concluded that JPG compression does not seem to significantly compromise the accuracy of angiogenesis quantification in ovarian epithelial tumors. In contrast, López et al.^34^ studied the effects of image compression on automatic quantification of immunohistochemical nuclear markers and found it to be dependent on the image content (number of cells per field and number/size of clusters)—the effect was small for low-complexity images (≥100 cells per field, without clusters or with small/area clusters) and substantial for high-complexity images (<35 to 50 cells/field). Overall, it is important to note that these reports largely differ in their content of images, characteristics of lesions, and diagnostic tasks under study (detection or quantification of lesions), as well as in the range of compression ratio/quality. This confirms again the importance of conducting application-specific IQA instead of making (under investigated) assumptions or generalizations from one case to another, as asserted in the introduction.

Next, we look at the M-JPG-related data across our experimental setups. Those rankings of image alterations do not always agree. First, staying within the same exp1, the pIQ ratings from $Q_{1}$ suggest significantly lower quality of M-JPG images compared to the reference M-NONE images [see Fig. 4(a)]. Thus, within exp1, the findings concerning M-JPG are in agreement between the two experimental tasks/questions, that is, cIQ ratings are in agreement with pIQ ratings. However, the findings of exp2 and exp3 about M-JPG are no longer in agreement. The similarity ratings from exp2 indicate high similarity between M-JPG and M-NONE (median [IQR]=4.5[4,5], see Table 7), suggesting that pathologists were not (much) noticing the JPG artifacts. Likewise, the pIQ ratings from $Q_{1}$ from exp3 rank M-JPG no different than M-NONE. In other words, there was something that the observers saw under exp1 (pIQ of M-JPG ranked lower than pIQ of M-NONE) but which they were missing under exp3 (no difference in pIQ of M-JPG and M-NONE) and even with exp2 (little dissimilarity between M-JPG and M-NONE).

That being so, it appears from our results that the pathologists’ answers to the question of “How would you judge the overall quality of the image?” were not always the same (see Fig. 4). The following two factors could be contributing to this effect: the observer’s instructions and the experiment context. First, we recall that in exp1 the pathologists were instructed to judge the overall quality of the images according to their own personal criteria, while in exp3 the observers received some training about the attributes of IQ (blur, noise, contrast, and color saturation). Therefore, it is possible that the training they received in exp3 led the pathologists to focus on some specific types of image impairments (artifacts), but, because they were otherwise little familiar with them, the task might have been distracting and/or confusing. Previously, Platiša et al.^18^ reported that in the exact same experiment setup performed by image-processing researchers, the pIQ was always ranked lower for M-JPG compared to M-NONE. Therefore, while JPG artifacts were obvious to the trained eye of the imaging expert, the pathologist seemed to lack the necessary skills to recognize the JPG as such but rather became aware of the degradation in quality only at the point where the diagnostic task was at stake (clinical experiment context in exp1). So could it be that the context (created by the actual clinical task) is affecting (helping) the overall IQ judgment? In light of the latest findings of the significant differences in viewing behavior (gaze response) in the task of rating quality versus free-looking at an image (no goal/task specified),^35^^,^^36^ the factor of the experimental context could be a perfectly valid candidate for future investigation. It could be that the context of the experiment deserves more research attention than it has been granted so far.

On the other hand, from the range of the rating scales that the pathologists used for pIQ (see Fig. 4), it seems that they were more critical of IQ in the clinical context when the true clinical need was at stake (pIQ ratings were largely in the lower half of the scale) unlike in the technical context when nothing “practical” was really gained or lost (pIQ ratings fall toward the mid and upper parts of the scale). Additionally, perhaps this less conservative use of the rating scale in exp3 was caused by seemingly better understanding of the concept of IQ (after the training in IQ and its attributes) compared to that in exp1 (no such training). Of course, we also note that the two rating scales differ in their nature—discrete in exp3 versus continuous in exp1. This in itself could be the topic for further discussion,^37^ yet it is beyond the scope of our consideration here.

Finally, the variation in the ratings of a single individual when judging the same image on multiple occasions (reproducibility) could be attributed to the well-known and much-studied effect of “intraobserver” variability. However, albeit a limited number of observers, the effect of a single observer can hardly explain the difference in the average performance of multiple observers, which occurs in our study.

Importantly, the data of our study suggest the necessity to define a clinical task and conduct task-based observer experiments (as in exp1) to assess cIQ of digital pathology data. Other considered experiment setups could disguise some of the diagnostic/clinical effects of the images and thus cannot be fully trusted as a means of assessing clinical image utility. Alternatively, in the cases where it may not be possible to involve actual conducting of a clinical task in the experiment, it is important to at least create the clinical context for the experiment to stimulate the observer’s awareness of the critical factors in the actual clinical environment (e.g., risk of misdiagnosis and risk of under/overtreatment.).

At the end of this section, we acknowledge the limitations of our study and highlight the key lessons learned; we then suggest some recommendations for similar studies in the future. The main limitation of our experiments is the small test image sample size (12 reference images) as well as the dependency between the reference and their corresponding altered images. As indicated in Sec. 3, the required number of cases for doing JAFROC random-case analysis would be above 50. Another related point for improvement is the variation in the test image content. Our current report does not investigate the effect of image content (tissue type) while that could play an important role in drawing conclusions,^15^ and thus it should be considered in future research. The measurements of exp3 grouped by tissue type can be found in Ref. 18.

In our experiments, only one level of each type of image alteration was selected based on the visual differences predicted by HDR-VDP^23^ measure. One major limitation of this approach is that at the time of our experiment the measure was not adjusted for color images; hence, the extent of our color-related alterations could be different from those of the spatial-related alterations (e.g., added noise and blur filtering). For future experiments with humans, we recommend replacing or amending the process of selecting the levels of image alterations in a pilot study with humans, following, for example, the method presented by Kumcu et al.^38^ Additionally, before any conclusions could be made about the (positive or negative) effect of a given type of image alteration on the quality of images, it will be necessary to include in the experiments multiple levels of a given type of image alteration.

Concerning image compression, next to the JPG considered in our experiments, it is also necessary to refer to JPEG2000, especially at higher compression ratios. Supplement 145 of the DICOM standards^39^ states the following concerning image data compression: “Because of their large size, WSI data are often compressed. Depending on the application, lossless or lossy compression techniques may be used. Lossless compression typically yields a 3X-5X reduction in size. The most frequently used lossy compression techniques are JPEG and JPEG2000. For most applications, pathologists have found that there is no loss of diagnostic information when JPEG [at 15X-20X reduction] or JPEG2000 [at 30X-50X reduction] compression is used. Lossy compression is therefore often used in present-day WSI applications. JPEG2000 yields higher compression and fewer image artifacts than JPEG; however, JPEG2000 is compute-intensive.”^40^ Similar to the studies of JPG compression, current literature reporting the effects of JPEG2000,^41^^,^^42^ albeit limited, suggest the need for future investigations to be directed at specific applications (anatomy, diagnostic task, and compression ratios of interest).

Related to the diagnostic task of detecting inclusion bodies, it is important to note that the FROC setup from our experiment is not used routinely in daily practice. Instead, in daily routine, solely the presence or absence (not actual number) of inclusion bodies is noted in the context of a range of other degenerative cell lesions and inflammatory lesions, supporting the histopathologic diagnostic conclusion. The FROC setup was chosen as an acceptable surrogate for the real clinical procedure with existing statistical tools for data analysis. For future studies involving the diagnostic task, it will be of interest to replicate the actual diagnostic routine as closely as possible to maximize experimental realism and to properly use pathologists’ skills in the process of image evaluation.

Finally, the work presented in this paper identifies several points of interest for further research. In particular, it would be important to better understand the role of the experimental context in the human observer studies. Next to guiding the design of future studies with humans, this might also give important insights for the future effort in developing numerical measures of IQ. For example, when collecting human data for the purpose of developing numerical IQ measures, it might be of interest to tie the questions about different quality attributes to the task of interest, perhaps by asking the observers to perform the clinical task and evaluate the quality of the data within the same context, similar to what was done in our exp1. In current research practice, these two types of questions—clinical task versus quality ratings—are commonly asked in different contexts (during separate experimental sessions). In fact, most often, only one type of question is considered, either a clinical task or the pIQ-attribute ratings. It would be of interest to see whether, by ensuring a clinical context for the experiment, the true clinical task could be replaced by simpler nonclinical tasks and still lead to the same conclusions. A related open question is the best experimental setup for leading the observers to consider the clinical relevance of the images without actually conducting a clinical task. Last, while exploring the effect of the context and the task/question, the rating scales for the same questions should be kept the same to allow proper comparison.

## 5 Conclusions

The aim of our study was to investigate the optimal methodology for IQA of digital pathology images and, more specifically, to examine possible interaction between the two common approaches of pIQ and cIQ. To the authors’ best knowledge, no literature reports to date have evaluated this relationship. Our experimental data were collected in three image reading experiments conducted to assess the impact of various image manipulations on the quality of digital veterinary pathology slides under the H&E staining as judged by veterinary pathologists. Exp1 was framed in a clinical context; it specified the task of detection and localization of inclusion bodies in the images, and it measured cIQ by means of observers’ FROC performance as well as task-aware pIQ through observers’ ratings of overall IQ. Exp2 asked the observers to rate pairs of images for their similarity and preference, and Exp3 again collected ratings of pIQ but in a nonclinical context.

Based on the data from exp1, the diagnostic performance as well as the ratings of overall IQ for the reference images decreased after the JPG compression was applied. Thus, when there was a specific clinical task defined, the pIQ and cIQ were in correlation. Nevertheless, the pIQ data that came from exp2 and exp3, which had no clinical task defined, did not always rank the image alterations the same as their diagnostic performance did.

The main contribution of our work is in presenting preliminary evidence that, for digital pathology images under study and for the considered clinical task, the results of two leading experimental strategies for IQA disagree, namely pIQ is not always correlated with cIQ. Moreover, we introduced a concept of task-aware pIQ. In that approach, the observers are rating the pIQ within a clinical context provided by specifying the target clinical task that evokes observers’ appraisal of the clinical relevance of the images but without conducting the task. Based on our results, this strategy appears to be a promising surrogate for performing the actual clinical task. Further research is needed to assess whether and for which purposes (e.g., preclinical testing) task-aware pIQ ratings could substitute cIQ for a given clinical task.
